# SARS-CoV-2 – a new challenge for laboratory medicine

**DOI:** 10.11613/BM.2020.030503

**Published:** 2020-08-05

**Authors:** Slavica Dodig, Ivana Čepelak, Daniela Čepelak Dodig, Renata Laškaj

**Affiliations:** 1 Department of Medical Biochemistry and Hematology, Faculty of Pharmacy and Biochemistry, University of Zagreb, Zagreb, Croatia; 2 Department for Toxicology, Croatian Institute of Public Health Zagreb, Croatia; 3 Department of Medical Biochemistry, Hematology and Coagulation, University Hospital for Infectious Diseases, Zagreb, Croatia

**Keywords:** biomarkers, COVID-19, SARS-CoV-2, laboratory medicine, patient safety

## Abstract

The new corona virus SARS-CoV-2 (Severe Acute Respiratory Syndrome Corona Virus 2) causes a disease called COVID-19 (coronavirus disease 2019), that develops mostly in subjects with already impaired immune system function, primarily in the elderly and in individuals with some chronic disease or condition. The reasons for this should be sought in the processes of aging and chronic latent inflammation, *i.e.* immunosenescence and inflammaging. Laboratory medicine specialists are currently focused on proving the presence of the virus and defining biomarkers that would enable the prediction of disease progression. For now, it has been shown that useful biomarkers can include general biomarkers of inflammation (parameters of complete blood count, C-reactive protein, interleukin-6, procalcitonin), biomarkers of myocardial damage (high sensitivity troponin I/T, B-type natriuretic peptide, and N-terminal B type natriuretic peptide), and vascular biomarkers (D-dimer, prothrombin time, fibrinogen). Their actual diagnostic specificity, sensitivity and predictive value need to be tested on a larger number of subjects. In addition, it is important to find and evaluate specific biomarkers of immunosenescence.

## Introduction

The novel beta-coronavirus named SARS-CoV-2 (Severe Acute Respiratory Syndrome Corona Virus 2), also known as *new CoV*, *2019-nCoV* or *COVI-19*, which appeared in China at the end of 2019, caused a novel pandemic disease defined as COVID-19 (coronavirus disease 2019) (*1*). To date, the detailed structure and physicochemical properties of this new coronavirus, as well as some clinical characteristics of COVID-19 are still unknown.

It has been shown that most patients have their first symptoms as early as the fifth day after infection. The incubation period ranges from 1 to 28 days, but on an average is 1-14 days. However, in some cases the incubation period of almost one month has been reported (*1*-*3*). Signs and symptoms of CoV-caused illness range from the common cold to more severe respiratory disease/syndrome. Common signs of COVID-19 include fatigue, muscle pain, fever, dry cough, sputum production, shortness of breath, dyspnoea, sore throat, and headache (*4*).

Short clinical experience with SARS-CoV-2 infection has so far shown that young people have mild symptoms, mainly in the upper respiratory tract. Moreover, in some individuals the infection is even asymptomatic. A small number of the described patients had headache, confusion, febrile seizures, convulsions, and some were diagnosed with encephalitis, which shows that the virus in these patients spread from the respiratory system to the central nervous system (*5*). Older age, and comorbidities such as diabetes, cardiovascular diseases, chronic respiratory diseases, renal disease, coagulopathy *etc.*, are reported as significant predictors of morbidity and mortality (*3*, *6*). The reason for such progression of disease severity should be sought in the processes of immunosenescence and age-associated chronic inflammation (*7*, *8*).

Timely, accurate and reliable laboratory detection of SARS-CoV-2 in respiratory tract specimens is crucial for early diagnosis of COVID-19. Because the virus can be detected by complex diagnostic procedures, it is important for the patient safety that all stages of the analytical process are performed according to the principles of good laboratory practice. In addition, in order to assess the risk of developing severe forms of the disease, it is certainly important to select biomarkers of both inflammation and aging, and to determine their predictive value in patients with COVID-19.

The aim of this short review is to present the literature data available at the time of writing this article, as well as recommendations for COVID-19 laboratory diagnostics, and monitoring of patients during disease development, with an emphasis on patient safety during all stages of the analytical process. In search of review and scientific papers on the PubMed free search engine, following key words were used: coronaviruses, SARS-CoV-2, COVID-19, immunogenicity, biomarkers, methods, immunosenescence, inflammation, and patient safety. Articles published in English between 2004 and June 2020 were included, and were selected according to relevance to the topic.

## Pathogenesis and immune response

So far, the pathogenesis of SARS-CoV-2 and the host immune response can be discussed based on knowledge of these processes in infections with other coronaviruses and viruses in general. SARS-CoV-2 is positive-sense single-stranded ribonucleic acid (RNA) virus, which contains four major structural proteins, *i.e.* the spike (S), membrane (M), envelope (E) and nucleocapsid (N) proteins, essential to produce a structurally complete viral particle and for infection (*9*-*11*). The spike (S) glycoprotein attaches the virion (*i.e.* virus outside the host cell) to the host cell membrane. SARS-CoV infects type 2 pneumocytes and ciliated bronchial epithelial cells utilizing angiotensin converting enzyme 2 (ACE2) as a receptor, and the N protein is involved in virus replication in the host cell (*12*, *13*).

The immune response to infection with SARS-CoV-2 takes place in two stages. Like other viruses, SARS-CoV-2 first replicates rapidly in epithelial cells of respiratory and enteric system. Thereafter, non-severe stage, *i.e.* non-pneumonia and mild pneumonia, begins, when innate immune mechanisms eliminate the virus and to prevent the disease progressing to the severe stage.

In order to successfully defend itself, the human organism must be of good general health and must have an appropriate genetic background (*e.g.* HLA, human leukocyte antigen) that has a significant effect on specific antiviral immunity (*14*). In individuals with impaired immunity, the virus will almost undisturbedly cause massive destruction of the target tissues, especially if the organs have significant ACE2 expression, such in the lungs, intestines, and kidney. Due to inflammation damaging, pneumonia is the main cause of life-threatening respiratory disorders in the severe stage of the disease (*15*).

According to some authors, there are three reasons for a serious, life-threatening condition in patients. These are: HLA haplotypes, cytokine storm (CS) and hyaluronan (HA) accumulation. Different HLA molecules affect cellular immunity, CS increases inflammation, and HA influences fibrotic processes (*14*). HLAs play an essential role in activation of natural killer cells and T cells (*16*). The development of a more severe form of the disease is affected by the host HLA-haplotype, since the polymorphic nature of HLA is significantly associated with the clearance of different viruses by cellular immunity. In addition, during severe stage, the production and regulation of HA is defective. During severe stage, there are increased levels of inflammatory cytokines interleukin-1 (IL-1), tumour necrosis factor (TNF) in the lungs of patients, which induce hyaluronan-synthase-2 (HAS2) and consequent accumulation of HA, which subsequently perpetuates inflammation and promotes fibrotic processes in the lungs (*17*). The release of numerous cytokines, which are involved in the immunopathological damage in patients, contributes to their severe condition.

## Laboratory testing

### Patient safety

Patient safety in the context of laboratory medicine involves the timely receipt of reliable results, which allow physicians, in the whole process of synthesizing clinical data and diagnostic procedures, to diagnose and plan further diagnostic and therapeutic procedures (*18*, *19*). Laboratory diagnostics includes three phases of the process - preanalytical, analytical and postanalytical phase, and all are crucial for patient safety. Therefore, avoiding sources of error during whole diagnostic procedure increases the degree of patient safety (*19*).

### Laboratory biosafety

Throughout the procedure, all staff need to be aware that each biological sample is potentially infectious. Therefore, it is extremely important that all medical and laboratory personnel involved in the diagnostic procedures of SARS-CoV-2 adhere strictly to the appropriate biosafety precautions and practices (*e.g.* medical protective clothing, gloves, eye protection - goggles or face shields, not personal eyeglasses or contacts, disinfectants) (*20*-*22*). Personnel must be well educated and trained, not only for specific analytical skills but also for safety procedures. Handling and processing all biological specimens (*i.e.* respiratory specimens, haematological, biochemical specimens used, for microbiological, non-culture-based diagnostic analyses, and polymerase chain reaction (PCR) analysis, haematological and biochemical analyses) have to be performed according to the good laboratory practices and procedures (*20*).

### Disinfectants

Because knowledge of SARS-CoV-2 is still insufficient, it is necessary to use disinfectants that have already been proven to be effective against other coronaviruses, *e.g.* Middle East respiratory syndrome (MERS) (*20*). Particular attention should be given to appropriate disinfectants. Such disinfectants must have proven activity against enveloped viruses, must act for the recommended contact time (10 minutes) and must not be used after expiry date after the working solution (of prescribed dilution) is prepared. The following biocidal agents can thus be used: 62-71% ethanol, 0.5% accelerated hydrogen peroxide, quaternary ammonium compounds, phenolic compounds (following the manufacturer’s recommendations). Other agents such as 0.05–0.2% benzalkonium chloride or 0.02% chlorhexidine digluconate may be less effective. In addition to these agents, sodium hypochlorite (*e.g.* 0.1% *i.e.* 1000 parts *per* million (ppm)) for general surface disinfection and 10,000 ppm (1%) for disinfection of blood spills) may also be used (*23*, *24*).

## Diagnostic procedures

For the purpose of epidemiologic surveillance, in areas with already spread SARS-CoV-2, two methods are carried out: confirmation of viral nucleic acids in respiratory tract specimens and after an appropriate time detection of specific SARS-CoV-2 antibodies in serum, respectively (*25*). As the basic diagnostic criterion for an infectious disease is the detection and confirmation of the cause of particular disease, laboratory diagnostics play a central role in the etiological diagnosis of SARS-CoV-2. The nucleic acid amplification testing (NAAT) in biological specimens obtained from respiratory tract by real-time PCR and further confirmed by next-generation sequencing, is the golden standard for the diagnosis of COVID-19 (*26*, *27*). Nucleic acid amplification testing of appropriate respiratory tract samples in patients with more critical conditions and for suspicious cases are recommended by the World Health Organisation (WHO) (*28*).

## Preanalytical phase

### Specimens

Specimens may be collected from upper respiratory tract (nasopharyngeal (NS) and oropharyngeal swab (OS) or nasal wash (NW)) in ambulatory patients. In addition to these samples, sputum (if produced) and/or endotracheal aspirate or bronchoalveolar lavage (BAL) should be collected from patients with more severe disease (*28*). Samples are collected and transported in commercially prepared tubes containing specific viral transport medium with antifugal and antibiotic supplements. Haematological and biochemical parameters are important for inpatients laboratory monitoring. In surviving patients, two serum specimens, *i.e.* acute and convalescent ones, may be useful to for the determination of specific antiviral immunoglobulin (Ig) M (indicator of recent infection) and IgG (indicator of convalescent phase) antibodies (Table 1). Autopsy material including lung tissue should be taken in case of patients who are deceased.

**Table 1:** Specimens to be collected from outpatients and inpatients

**Patient**	**Sample**	**Laboratory test**	**Timing**
Contact (outpatients)*	NS, OS	NAAT	During incubation period of last documented contact
	Serum (paired samples)	Antiviral antibodies (IgM, IgG)	Baseline value within incubation, and convalescent serum 2-4 weeks after last contact.^†^
**Inpatients**	Sputum, aspirate, BAL, NS, OS, NW,	NAAT	Collected on presentation; possibly repeated to monitor clearance.^‡^
	Blood, serum	Haematological and biochemical, coagulation, antibodies (paired samples), inflammatory markers	*E.g.* CBC, enzymes, urea, creatinine, CRP, D-dimers, antiviral antibodies, interleukins, ABS, blood gasses
*Contacts have symptoms, or asymptomatic contacts have had high-intensity contact with COVID-19. ^†^Optimal timing for convalescent sample needs to be established. ^‡^Further research needs to determine effectiveness and reliability of repeated sampling. NS – nasopharyngeal swab. OS - oropharyngeal swab. NW - nasopharyngeal wash. BAL - bronchoalveolar lavage. NAAT - nucleic acid amplification test. CBC - complete blood count. CRP - C-reactive protein. ABS - acid-base status. IgG - immunoglobulin G. IgM - immunoglobulin M. Adapted according to reference 28.			

### Specimens’ storage and transport

Specimens for SARS-CoV-2 detection should reach the laboratory as soon as possible after collection. Storage and transport temperature of specimens should be maintain between 2 and 8°C. At this temperature, NS and OS can be stored for ≤ 5 days and sputum and BAL for ≤ 2 days. Otherwise, these samples should immediately be frozen at - 70°C. Post-mortem tissue biopsies can be stored at 2–8°C for ≤ 24 h, or at 70°C for a longer period. Repeated freezing and thawing of specimens should be avoided (*29*).

## Analytical phase

### Detection of SARS-CoV-2

Mucoid or mucopurulent material that make the sputum specimen too viscous must be removed from the specimen before detection of SARS-CoV-2. The virus detection procedure involves three stages: (i) virus extraction, (ii) PCR amplification and (iii) confirmation by nucleic acid sequencing when necessary. Currently, the *N, E, S* and *RdRP* viral genes are targeted (*28*). In the first (screening) procedure that detects the E gene, a nucleic acid amplification PCR test is required. Confirmation of SARS-CoV-2 is based on detection of unique sequences of virus RNA by NAAT such as real-time reverse transcription polymerase chain reaction (rRT-PCR) followed with confirmation by nucleic acid sequencing when necessary (*26*-*28*, *30*).

Laboratory personnel seeking to introduce detection method of SARS-CoV-2 should already have experience with the PCR method. Moreover, the SARS-CoV-2’s genes detection test should be performed in laboratories already accredited for PCR technology. All procedures in biosafety level-2 cabinets must meet quality standards such as ISO 15189:2012 or EN 12469 (*31*, *32*). Quality control is performed with internal and external control samples. For internal control, three samples are used: negative control, positive control and inhibition control to detect PCR inhibitory substances. Positive patient findings are confirmed by repeating the PCR using the original sample, or by confirmation in a second laboratory. The specificity of the procedure can be increased by amplifying a second genome region (*16*, *17*, *28*).

## Postanalytical phase

Knowledge of the circulation of a virus in an area may help to interpret the results obtained. Namely, it is important to know whether patients originate from areas with established SARS-CoV-2 circulation or subjects live in areas with no known virus circulation. It is considered that in areas with widespread virus, detection by rRT-PCR of a single discriminatory target is sufficient (*28*). However, one or more negative results does not rule out a possible infection. False negative results in infected patients can be found for several reasons: if specimens were not handled and transported appropriately; due to poor quality of specimen (insufficient amount of material for the PCR testing); if specimens were collected very early or late in the infection; due to PCR inhibition; because of possible mutation of virus. The PCR testing should be repeated if a negative result is obtained in patient with a high index of suspicion for SARS-CoV-2 infection, especially if the first sample was from the upper respiratory tract. In this case, the sample from the lower respiratory tract should be analysed (*28*).

In areas with no SARS-CoV-2 circulation, one of the following conditions need to be met: (i) a positive NAAT result for at least two different targets on the SARS-CoV-2 genome, of which at least one target is preferably specific for virus (when validated assay was used), and (ii) one positive result of NAAT for the presence of beta-coronavirus and SARS-CoV-2, further identified by sequencing partial or whole genome of the virus, as long as the sequence target is larger or different from the amplicon probed in the used NAAT (*25*, *28*, *33*).

If discordant results are obtained, the patient should be resampled, and repeated sequencing of the virus from the original specimen or of an amplicon generated from an appropriate NAAT assay, different from the NAAT assay initially used - should be obtained to provide a reliable test result (performed in another reference laboratory). Sensitivity of PCR method can be increased if multiple specimens (*e.g.* NS, OS, *etc.*) are analysed. Specificity is excellent if quality control guidelines are applied. False positive results, which may occur as a result of technical errors (possible contamination), can be avoided if every positive test is verified (*34*).

## Laboratory monitoring of patients: haematological and biochemical parameters

According to recent literature data, COVID-19 patients have changed values of non-specific haematological and biochemical parameters, depending on the severity of the disease (*12*, *25*, *35*). Available data indicate that in the early stage of the disease, most patients have a normal or decreased leukocyte count with lymphocytopenia (due to a decreased immune response to virus); unchanged/increased monocyte count; increased activity of lactate dehydrogenase (LDH) – as a marker of pulmonary injury, aminotransferase (AST) or alanine aminotransferase (ALT) above the upper limit of the reference range (indicators of liver injury and/or widespread organ damage) (*12*, *35*). Non-specific inflammatory factors are also increased, *e.g*. C-reactive protein (CRP), as well as ferritin, IL-6, IL-10, TNF-α, correlating with the severity of the illness (*4*). The procalcitonin concentration is within reference intervals, but is increased in patients with secondary infections (*12*). Lymphopenia is a negative prognostic factor. However, patients with serious condition have neutrophilia (simultaneously lymphocyte counts continued to decrease), significantly higher values of urea, creatinine, amylase and D-dimer, as a sign of multiorgan imbalance. After initial tissue damage, in some patients SARS-CoV-2 may induce exaggerated production of proinflammatory cytokines, the recruitment of proinflammatory macrophages and granulocytes. These processes result in the development of a macrophage activation syndrome or secondary haemophagocytic lymphohistiocytosis, with release of numerous cytokines, a condition called CS (Table 2) (*36*, *37*). Patients treated in hospitals and especially patients in intensive care units have an even more pronounced increase in the concentrations of numerous proinflammatory cytokines and chemokines (*4*, *38*). The severity of the disease correlates with the parameters of cellular immunity in the blood and serum concentrations of proinflammatory cytokines, and CS inevitably leads to acute respiratory distress syndrome (ARDS) (*38*-*40*). Hyperinflammation, therefore, worsens the prognosis of COVID-19.

**Table 2:** Trend of variations over time of laboratory parameters related to inflammation

**Phase of disease**	**Serum cytokines and chemokines profiles**	**Blood and serum parameters related to inflammation**
**Initial phase**	() IL-1β, IL-1RA, IL-7, IL-8, IL-10, IFN-ɣ, MCP-1, MIP-1A, MIP-1B, G-CSF, TNF-α	() leukocytes, neutrophils, monocytes (Ż) eosinophils () acute phase proteins
**Worsening of the disease**	() IL-2, IL-6, IL-8, IL-10, TNF-α	() lymphocytes, monocytes, platelets, N/L, M/L (Ż) eosinophils () CRP, D- dimer, fibrinogen, ferritin
**Non-ICU patients**	() IL-2, IL-7, IL-17, IL-10, IP-10, MCP-1, MIP-1A, TNF-α	() lymphocytes, monocytes, eosinophils, platelets, N/L, M/L, P/L () D-dimer, fibrinogen ferritin, procalcitonin
**ICU patients**	() IL-2, IL-6, IL-8, IL-10, TNF-α (higher than in non-ICU)	(Ż) lymphocytes, monocytes, eosinophils, platelets () N/L, D-dimer, fibrinogen, ferritin, procalcitonin
CRP – C-reactive protein. G-CSF – granulocyte-colony stimulating factor. ICU – intensive care unit. IFN – interferon. IL – interleukin. MCP – monocyte chemoattractant peptide. IP-10 – 10 kDa IFN γ-induced protein. MIP – macrophage inflammatory protein. M/L – monocyte/lymphocyte ratio. N/L – neutrophil/lymphocyte ratio. P/L – platelet/lymphocyte ratio. TNF-α – tumour necrosis factor-alpha. Adapted according to references 37-40.		

Gao Y. *et al.* wanted to find out which of the haematological and biochemical indicators had a good discriminatory value to distinguish the more severe cases of COVID-19 from mild cases (*41*). According to their results, glucose, CRP, IL-1, thrombin time (TT), fibrinogen and D-dimer differed significantly depending on the severity of clinical symptoms. IL-6, D-dimers, glucose, TT and fibrinogen had the best diagnostic significance (diagnostic specificity and sensitivity).

In patients with sepsis, haematological findings indicate thrombocytopenia and coagulopathy, while biochemical findings indicate the presence of acidosis, high lactate values and hyperbilirubinemia. In the case of septic shock the lactate value is greater than 2 mmol/L, as evidence of cellular/metabolic abnormalities (*12*).

Recently, laboratory medicine specialists have focused on determination of diagnostic sensitivity and specificity, and the predictive value of already known biomarkers of inflammation, biomarkers of damage to individual organs, and the identification of new, more reliable biomarkers. Moreover, since the elderly are at risk of a more severe form of COVID-19, it is necessary to select possible biomarkers of aging. So far, most attention has been paid to general biomarkers of inflammation (parameters of complete blood count, CRP, IL-6, procalcitonin), biomarkers of myocardial damage, *e.g.* high sensitivity troponin I/T (indicators of cardiomyocyte injury), B-type natriuretic peptide (BNP), and N-terminal B type natriuretic peptide (NT-proBNP) (indicators of haemodynamic stress), vascular biomarkers (D-dimer, prothrombin time, fibrinogen). D-dimers, as a prominent feature in COVID-19, may be used as an indicator of thrombin formation, unspecific inflammation and possible disseminated intravascular coagulation (*41*-*43*). Their concentrations correlate with disease severity and mortality. In addition to these known parameters, the value of other possible biomarkers (*e.g.* eosinophil count, serum concentrations of amyloid protein A, myeloperoxidase, ferritin, uric acid) is likely to be examined. Increased uric acid is an indicator of various processes in severe lung diseases (*e.g.* tissue hypoxia, anti-oxidative and pro-oxidative processes, inflammation-stimulatory effect) (*44*). Age, the presence of secondary infection, the presence of comorbidities, and inflammatory biomarkers were examined as predictors of mortality (*45*). It would be useful to define a pattern with a few cytokines, as it would probably have a better diagnostic and predictive value than a single cytokine. It can be expected that scientists will also continue research into biomarkers of senescence and aging, *e.g.* telomere length, senescence-associated secretory phenotype (SASP) factors and senescence-associated beta-galactosidase (SA-β-GAL) (*46*).

Specific IgM and IgG against SARS-CoV-2 start to increase in serum about 10 days after symptom onset. In most patients seroconversion occurs within three weeks (*47*). This information is not only important for the individual, but also for the retrospective assessment of the attack rate or extent of an outbreak. These specific antibodies against the S and N antigens of SARS-CoV-2 can be determined by rapid immunochromatographic methods, enzyme immunoassay (EIA), fluorescence immunoassays (FIA) or chemiluminescence immunoassays (CLIA). There is still not enough reliable data in the professional literature on their analytical sensitivity and specificity. Also, it is not yet known how long these antibodies persist in the patient’s serum after infection, so the clinical value of serological tests is still under evaluation (*48*).

## Concluding remarks

Many aspects of SARS-CoV-2 and COVID-19 are still not understood. Further research is needed on both the detailed structure of the SARS-CoV-2 and the immunity-related studies to understand the pathogenesis. This would help to apply an appropriate therapeutic approach, and to improve disease prognosis.

For now, in clinical and diagnostic contexts, as well as in laboratory diagnostics, experts rely on the knowledge gained about already known coronaviruses and the diseases they have caused. On the other hand, publishing work for objective reasons is associated with different limitations (limited data, different reporting styles, different therapeutic approaches, different estimates of transmission time, *etc.*), which may be the reason for underestimating some data. It is difficult to make significant concluding remarks as new insights into the new SARS-CoV-2 change almost daily.

It is still considered that virus detection in the respiratory tract specimens by PCR technique is the only correct method to prove SARS-CoV-2. Scientists will need to pay attention to the proportion of positive cases that requires sequencing and monitor possible mutations that might affect the results of molecular testing.

It would be important to find out how much the increased concentrations of particular biomarkers (*e.g.* ferritin, uric acid) caused by SARS-CoV-2 infection and how much are the result of associated diseases. These dilemmas will be able to be resolved in comparative studies in otherwise healthy patients and in elderly patients with comorbidities that may affect certain laboratory parameters.

Specialists in laboratory medicine must adopt the latest insights into COVID-19, follow up-to-date methods on the development and validation of useful serological testing, as well as comparative study of available molecular and serological testing.

A quick and reliable diagnostic process will facilitate other specific public health interventions that will also be helpful in establishing timely and appropriate therapy. The main criterion to ensure the patient safety during laboratory diagnostics is to avoid performing non-specific and non-sensitive analyses and to obtain unreliable results, which could guide physicians to reach the wrong conclusions. The prevention of unnecessary burden of laboratory personnel as well as avoiding unnecessary expenses should also be kept in mind.
